# Acquiring and preprocessing leaf images for automated plant identification: understanding the tradeoff between effort and information gain

**DOI:** 10.1186/s13007-017-0245-8

**Published:** 2017-11-08

**Authors:** Michael Rzanny, Marco Seeland, Jana Wäldchen, Patrick Mäder

**Affiliations:** 1 Department Biogeochemical Integration, Max-Planck-Institute for Biogeochemistry, Hans-Knöll-Str. 10, 07745 Jena, Germany; 2 Institute for Computer and Systems Engineering, Technische Universität Ilmenau, Helmholtzplatz 5, 98693 Ilmenau, Germany

**Keywords:** Leaf image, Image acquisition, Preprocessing, Segmentation, Cropping, Background, Leaf side, Back light, Effort, CNN, Computer vision

## Abstract

**Background:**

Automated species identification is a long term research subject. Contrary to flowers and fruits, leaves are available throughout most of the year. Offering margin and texture to characterize a species, they are the most studied organ for automated identification. Substantially matured machine learning techniques generate the need for more training data (aka leaf images). Researchers as well as enthusiasts miss guidance on how to acquire suitable training images in an efficient way.

**Methods:**

In this paper, we systematically study nine image types and three preprocessing strategies. Image types vary in terms of in-situ image recording conditions: perspective, illumination, and background, while the preprocessing strategies compare non-preprocessed, cropped, and segmented images to each other. Per image type-preprocessing combination, we also quantify the manual effort required for their implementation. We extract image features using a convolutional neural network, classify species using the resulting feature vectors and discuss classification accuracy in relation to the required effort per combination.

**Results:**

The most effective, non-destructive way to record herbaceous leaves is to take an image of the leaf’s top side. We yield the highest classification accuracy using destructive back light images, i.e., holding the plucked leaf against the sky for image acquisition. Cropping the image to the leaf’s boundary substantially improves accuracy, while precise segmentation yields similar accuracy at a substantially higher effort. The permanent use or disuse of a flash light has negligible effects. Imaging the typically stronger textured backside of a leaf does not result in higher accuracy, but notably increases the acquisition cost.

**Conclusions:**

In conclusion, the way in which leaf images are acquired and preprocessed does have a substantial effect on the accuracy of the classifier trained on them. For the first time, this study provides a systematic guideline allowing researchers to spend available acquisition resources wisely while yielding the optimal classification accuracy.

## Background

Accurate plant identification represents the basis for all aspects of related research and is an important component of workflows in plant ecological research. Species identification is essential for studying the biodiversity richness of a region, monitoring populations of endangered species, determining the impact of climate change on species distributions, payment of environmental services, and weed control actions [1, 2]. Accelerating the identification process and making it executable by non-experts is highly desirable, especially when considering the continuous loss of plant biodiversity [3].

More than 10 years ago, Gaston and O’Neill [4] proposed that developments in artificial intelligence and digital image processing could make automated species identification realistic. The fast development and ubiquity of relevant information technologies in combination with the availability of portable devices such as digital cameras and smartphones results in a vast number of digital images, which are accumulated in online databases. So today, their vision is nearly tangible: that mobile devices are used to take pictures of specimen in the field and afterwards to identify their species.

Considerable research in the field of computer vision and machine learning resulted in a number of studies that propose and compare methods for automated plant identification [5–8]. The majority of studies solely utilize leaves for identification, as they are available for examination throughout most of the year and can easily be collected, preserved and photographed, given their planar nature. Previous methods utilize handcrafted features for quantifying geometric properties of the leaf: boundary and shape as well as texture [9–13]. Extracting such features often requires a preprocessing step in order to distinguish the leaf from the background of the image, i.e., a binary segmentation step. For the ease of accurate and simple segmentation, most studies use leaf images with a uniform, plain background, e.g., by utilizing digital scanners or photographing in a controlled environment [14]. Only few studies addressed the problem of segmenting and identifying leaves in front of cluttered natural backgrounds [15, 16].

At the same time, machine learning techniques have matured. Especially, deep learning convolutional neural networks (CNNs) have almost revolutionized computer vision in the recent years. Latest studies in object categorization demonstrate that CNNs allow for superior results compared to state of the art traditional methods [17, 18]. Current studies on plant identification utilize CNNs and achieve significant improvements over methods developed in the decade before [19–22]. Furthermore it was empirically observed that CNNs trained for a task, e.g., object categorization in general, also achieve exceptional results on similar tasks after minor fine-tuning (transfer learning) [18]. Making this approach usable in an experimental setting, researchers demonstrated that using pre-trained CNNs merely for feature extraction from images results in compact and highly discriminative representations. In combination with classifiers like SVM, these CNN derived features allow for exceptional classification results especially on smaller datasets as investigated in this study [17].

Despite all improvements in transfer learning, to successfully train a classifier for species identification requires a large amount of training data. We argue that the quality of an automated plant identification system crucially depends not only on the amount, but also on the quality of the available training data. While funding organizations are willing to support research into this direction and nature enthusiasts are helpful by contributing images, these resources are limited and should be efficiently utilized. In this paper, we explore different methods of image acquisition and preprocessing to enhance the quality of leaf images used to train classifiers for species identification. We ask: (1) How are different combinations of image acquisition aspects and preprocessing strategies characterized in terms of classification accuracy? (2) How is this classification accuracy related to the manual effort required to capture and preprocess the respective images?

## Methods

**Fig. 1:**
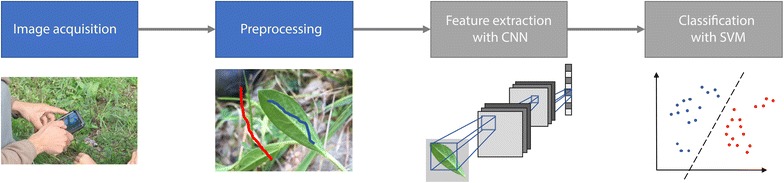
Consecutive steps of our research framework

Our research framework consists of a pipeline of four consecutive steps: image acquisition, preprocessing, feature extraction, and training of a classifier as shown in Fig. 1. The following subsections discuss each step in detail and especially refer to the variables, image types and preprocessing strategies that we studied in our experiments. We used state of the art feature extraction and classifier training methods and kept them constant for all experiments.

### Image acquisition

**Fig. 2:**
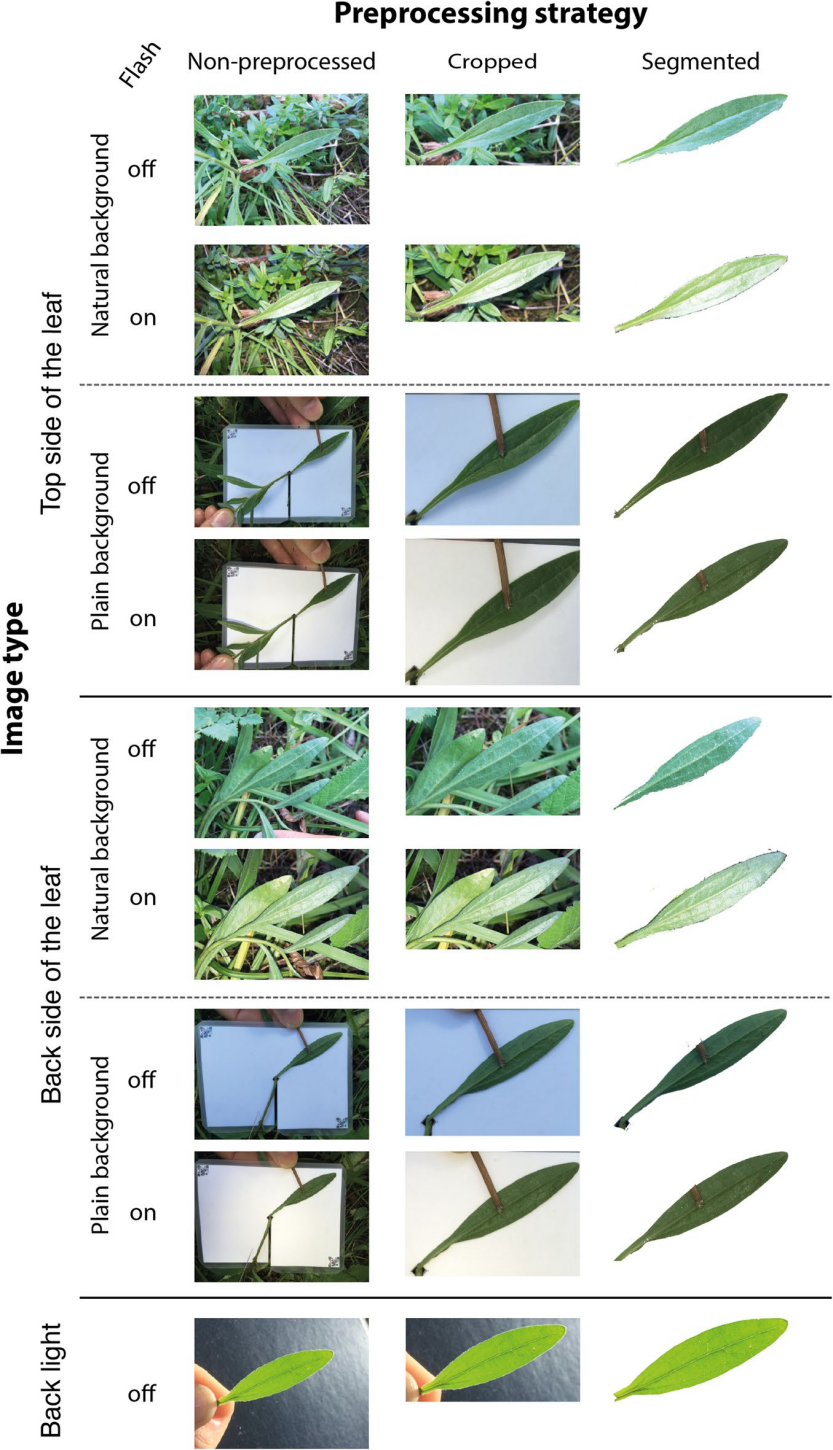
Leaf image set belonging to one observation of Aster amellus depicting all nine image types and the preprocessing strategies explored in this study

For each observation of an individual leaf, we systematically varied the following image factors: perspective, illumination, and background. An example of all images collected for a single observation is shown in Fig. 2. We captured two **perspectives** per leaf in-situ and in a nondestructive way: the *top side* and the *back side*, since leaf structure and texture typically substantially differ between these perspectives. If necessary, we used a thin black wire to arrange the leaf accordingly. We recorded each leaf under two **illumination** conditions using a smartphone: *flash off* and *flash on*. Flash off refers to a natural illumination, without artificial light sources. In case of bright sunlight, we used an umbrella to shade the leaf against strong reflections and harsh shadows emerging from the device, the plant itself, or the surrounding vegetation. Flash on was used for a second image, taken in the same manner, but with the built-in flashlight activated. We also varied the **background** by recording an initial image in the leaf’s environment composed of other leaves and stones, termed *natural background*. Additionally, we utilized a white sheet of plastic to record images with *plain background*. Leaves were not plucked for this procedure but arranged onto the sheet using a hole in the sheet’s center. Eventually, the leaf was picked and held up against the sky using a black plastic sheet as background to prevent image overexposure. This additional image type is referred to as *back light*. In summary, we captured nine different image types per observation.

All images were recorded with the use of an iPhone 6, between April and September 2016, throughout a single vegetation season. Following a strict sampling protocol for each observation, we recorded images for 17 species representing typical, wild-flowering plants that commonly occur on semi-arid grasslands scattered around the city of Jena, located in eastern Germany. At the time of image acquisition, every individual was flowering. The closest focusing distance represented a technical limit for the resolution of smaller leaves while ensuring to capture the entire leaf on the image. The number of observations per species ranged from eleven (*Salvia pratensis*) to 25 (*Pimpinella saxifraga)*. In total, we acquired 2902 images. The full dataset including all image annotations is freely available from [23].

### Image preprocessing

Each leaf image was duplicated twice to execute the three preprocessing strategies: **non-preprocessed**, **cropped**, and **segmented**. Non-preprocessed images were kept unaltered. Cropping was performed based on a bounding box enclosing the leaf (see Fig. 2). To facilitate an efficient segmentation, we developed a semi-automated approach based on the GrabCut method [24]. GrabCut is based on iterated graph cuts, and was considered accurate and time-effective for interactive image segmentation [25, 26]. The first iteration of GrabCut was initialized by a rectangle placed at the relevant image region, the focus area defined during image acquisition and available in an image’s EXIF data. This rectangle should denote the potential foreground whereas the image corners were used as background seeds. The user was then allowed to iteratively refine the computed mask by adding markers denoting either foreground or background, if necessary. The total amount of markers was logged for every image. To speed up the segmentation process, every image was resized to a maximum of 400 px at the longest side while maintaining the aspect ratio. Finally, the binary mask depicting only the area of the leaf was resized to the original image size. The boundary of the upsized mask was then smoothed using a colored watershed variant after morphological erosion of the foreground and background labels, followed by automated cropping to that mask.

### Quantifying manual effort

Image acquisition and preprocessing require substantial manual effort depending on the image type and preprocessing strategy. We aim to quantify the effort for each combination in order to facilitate a systematic evaluation and a discussion of their resulting classification accuracy in relation to the necessary effort.

For a set of ten representative observations, we measured the time in seconds and the amount of persons needed for the acquisition of each image. This was done for all combinations of the image factors **perspective** and **background**. Whereas a single photographer is sufficient to acquire images in front of natural background, a second person is needed for taking images with plain background and for the back light images in order to arrange the leaf and the plastic sheet. We then quantified the effort of image acquisition for these combinations by means of average ’person-seconds’ by multiplying the time in seconds with the amount of persons.

In order to quantify the manual effort during preprocessing, we measured the time in seconds an experienced user requires for performing either **cropping** or **segmentation** on a set of 50 representative images. For each task, the timer was started the moment the image was presented to the user and was stopped when the user confirmed the result of his task. For cropping, the time needed for drawing a bounding box around the leaf. This required 6.8 s on average independently from the image conditions. Image segmentation on the other hand involved substantial manual work depending on the leaf type, e.g., compound or pinnate leaves, and the image background. In case of natural background, often multiple markers were required. We measured the average time for setting one marker, amounting to 4.7 s, followed by multiplying this average time with the amount of markers needed for segmenting each image. In case of plain background and simple leaves, the automatically initialized first iteration of the segmentation process often delivered accurate results. In such cases, the manual effort was taken only to confirm the segmentation result, which took about 2 s. For compound and pinnate leaves, e.g., of *Centaurea scabiosa*, the segmentation task was considerably more difficult and required 135 s on average per image with natural background.

**Fig. 3:**
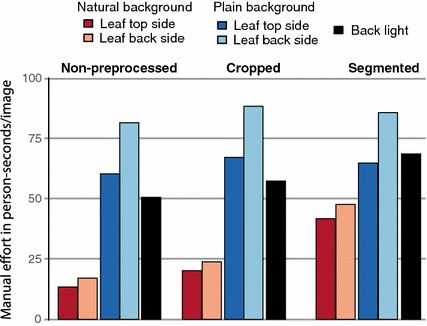
Mean manual effort per image, quantified by means of ’person-seconds’ for the five different image types and three preprocessing strategies

The mean effort measured in “person-seconds” for all combinations of image types and preprocessing steps is displayed in Fig. 3. We define a baseline scenario for comparing the resulting classification accuracy in relation to the necessary effort for each combination: With an empirically derived average time of 13.4 s, the minimum manual effort is in acquiring a top side leaf image with natural background and no preprocessing steps.

### Feature extraction

Using CNNs for feature extraction results in powerful image representations that, coupled with a Support Vector Machine as classifier, outperform handcrafted features in computer vision tasks [17]. Accordingly, we used the pre-trained ResNet-50 CNN, that ranked among the best performing networks in the ImageNet Large Scale Visual Recognition Challenge in 2015 [27], for extracting compact but highly discriminative image features. Every image was bilinearly resized to fit 256 px at the shortest side and then a center crop of 224$\times$224 px was forwarded through the network using the Caffe deep learning framework [28]. The output of the last convolutional layer (fc5) was extracted as 2048 dimensional image feature vector, followed by *L*2-normalization.

### Image classification

We used the CNN image features discussed in the previous section to train linear Support Vector Machine (SVM) classifiers. Each combination of the nine image types and the three preprocessing strategies resulted in one dataset creating 27 in total. These datasets were split into training (70% of the images) and test sets (30% of the images). In order to run comparable experiments, we enforced identical observations across all 27 datasets, i.e., for all combinations of image types and preprocessing strategies, the test and train sets were composed of the same individuals. Using the trained SVM, we classified the species for all images of each test dataset and calculated the classification accuracy as percentage of correctly identified species. All experiments were cross-validated using 100 randomized split configurations. Similarly, we quantified the species specific accuracy as percentage of correctly identified individuals per species. We used R version 3.1.1 [29] with the packages e1071 [30] for classifier training along with caret [31] for tuning and evaluation.

## Results

**Fig. 4:**
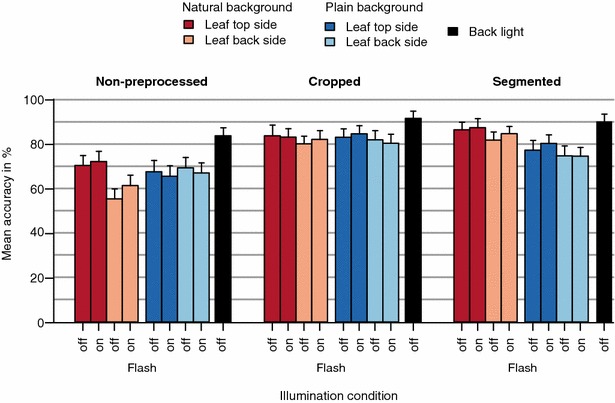
Classification results for different image types and preprocessing strategies, averaged across all species. Each bar displays the mean accuracy averaged across 100 randomized split configurations and the error bars display the associated standard deviation

Figure 4 displays the mean species classification accuracy separated for the nine image types and aggregated for the three preprocessing strategies. The highest classification accuracy ($91\pm 3$)% was achieved on cropped back light images, while the lowest accuracy was obtained for non-preprocessed backside images with natural background and without flash ($55\pm 4$)%. Across all three preprocessing strategies, back light images achieved the highest classification accuracy. Preprocessed images, i.e.cropped and segmented images, yielded higher accuracy than non-preprocessed images. Images of leaves in front of natural background benefit most from cropping and segmentation as they indicate the highest relative increase among the three preprocessing strategies. Starting with an already high accuracy, its relative gain between non-preprocessed and preprocessed back light images is smaller than that of natural and plain background images. Taking images with or without flash light seems not to affect classification accuracy in a consistent manner.

**Fig. 5:**
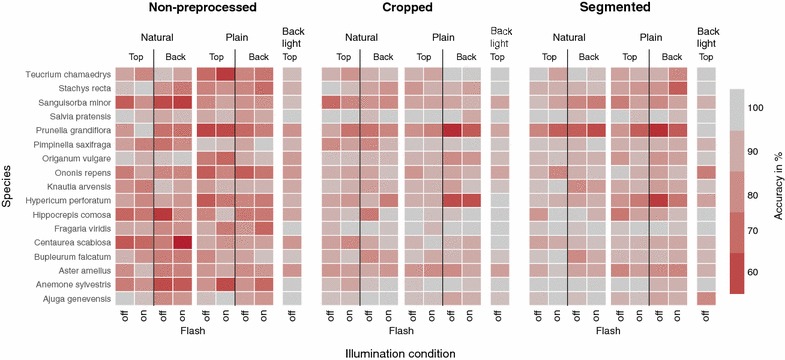
Mean classification accuracy averaged across 100 dataset splits. The accuracy for each combination of species and image type is color coded and aggregated per preprocessing strategy

Figure 5 shows classification results not only separated per image type and preprocessing strategy, but additionally per species within the dataset. The results show that classification accuracy depends on the classified species. While cropping and segmentation notably increase classification accuracy for some species, e.g., *Fragaria viridis* and *Centaurea scabiosa*, other species remain at a low classification accuracy despite of preprocessing, e.g., $\approx 70\%$ for *Prunella grandiflora*. Especially for these low-performing species, back light images yield a considerably higher accuracy, e.g., $>90\%$ for *Prunella grandiflora*. Furthermore, Fig. 5 shows that: (1) back light images yield a higher and more homogenous classification accuracy across the different species; (2) preprocessing by cropping or segmentation increases accuracy; and (3) the images with plain background achieve higher accuracies if cropped, while the images with natural background obtain higher accuracies when segmented prior to classification.

**Fig. 6:**
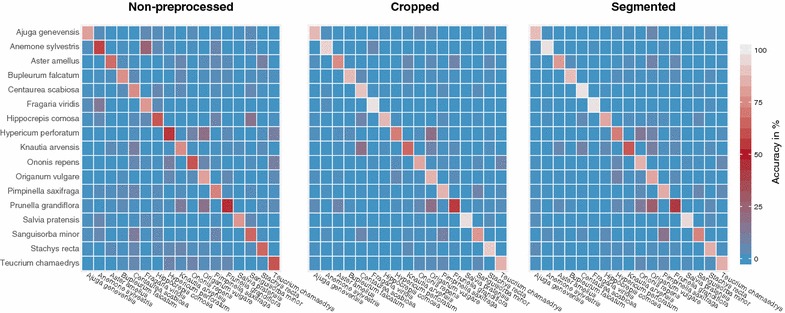
Confusion matrices presenting classification accuracy per preprocessing strategy. Observed species (rows) versus predicted species (columns) are averaged across the different image preprocessing steps

In order to investigate species dependent misclassifications in more detail, we computed the confusion matrices shown in Fig. 6. This figure illustrates the instances of an observed species in rows versus the instances of the same species being predicted in columns, averaged across all image conditions. We present one matrix per preprocessing strategy, visualizing how a certain species was confused with others, if its accuracy was below $100\%$. This is, for example, the case for *Anemone sylvestris* versus *Fragaria viridis* and *Prunella grandiflora* versus *Origanum vulgare*. Evidently, cropping and segmentation, notably decrease the sparse tendency towards false classification. *Prunella grandiflora* achieved the lowest classification accuracy across all species (Fig. 6) and was often misclassified as *Origanum vulgare*, a species with similarly shaped leaves. Some species, such as *Aster amellus* or *Origanum vulgare* are more or less invariant to subsequent preprocessing. Other species, such as *Fragaria viridis*, *Salvia pratensis*, or *Ononis repens* show very different results depending on whether or not the images were preprocessed.

These differences seem to be strongly species specific, even perspective specific without any discernible pattern. As an example, the classification accuracy of *Sanguisorba minor* is greatly improved by cropping for the backsides in front of natural background, while segmenting of these images does not further increase the classification accuracy (Fig. 5). In contrast, the accuracy of the topsides, also recorded in front of natural background is only slightly improved by cropping, while subsequent segmentation clearly improves the result. This species is a plant with pinnate compound leaves and the leaflets are often folded inwards along the midrib.

## Discussion

We found the classification accuracy to differ substantially among the studied image types, the applied preprocessing strategy, and the studied species. While species specific effects exist (see Figs. 5, 6), they were not the focus of our study and are not changing the general conclusions drawn from these experiments. The overall achieved classification accuracy is rather low when compared to other studies classifying leaf image datasets. In contrast to most other studies, our dataset comprises smartphone images of leaves from herbaceous plants. Here, small and varying types of leaves occur on one the same species. Many of the other datasets achieving higher accuracies primarily contain images of tree leaves that are comparably larger and can be well separated by shape and contour [6]. In addition, the leaves in our dataset were still attached to the stem upon imaging (except for the backlight images) and could not be perfectly arranged as it is possible for scanned, high resolution images in other datasets.

**Fig. 7:**
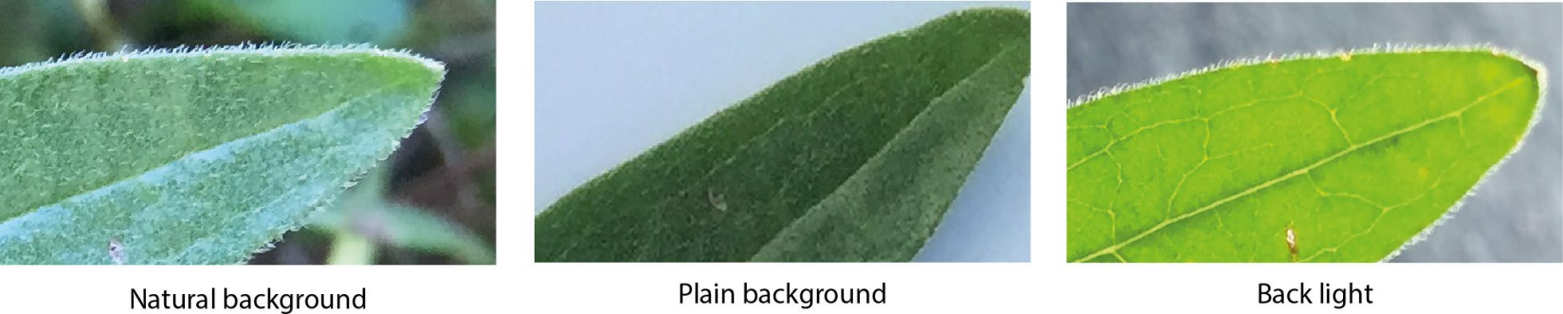
Detailed view of the leaf margins of the top side of *Aster amellus*. The images are the same as in the example shown in Fig. 2

We studied nine **image types** varying the factors: perspective (top side, back side, and back light), illumination (flash on, flash off), and background (natural and plain). Studying results for the different perspectives, we found that back light images consistently allowed for the highest classification accuracy across species and preprocessing strategies; followed by top side images that yield in most combinations higher accuracy than back side images, especially for the non-processed ones. Taking back sides images of a leaf, still attached to the stem, requires to bend it upwards while forcing it into an unnatural position. This results in variation across the images with respect to exact position, angle, focal plane and perspective, each hardly to control under field conditions. We studied illumination by enforcing a specific flash setting on purpose and found that it did not affect classification results in a consistent way. Hence, we conclude that automatic flash settings depending on the overall illumination of the scene may be used without negative impact on the classification result. However, in contrast to back light images, the different illumination conditions cause strong and undesired variations in the image quality. For example, a disabled flash results in images with small dynamic range while an enabled flash affects the coloring and creates specularities that mask leaf venation. We also varied the background by imaging leaves with plain—as well as natural background, of which the latter allowed for higher classification accuracy compared to plain background images. We found that imaging leaves in front of a plain background strongly affects the dynamic range of the leaves’ colors. Leaves in front of a plain background typically appear darkened and with an overall reduced contrast (cp. Figs. 2, 7).

We conclude from the analysis of image types that back light images contain the largest amount of visual information and the least amount of clutter (cp. Fig. 7). This image type facilitates: (1) sharply imaged leaf boundaries, especially in comparison to images with natural background; (2) homogeneously colored and illuminated leaves with high dynamic range making even slight details in the venation pattern and texture visible; (3) a lighting geometry that suppresses specularities by design; and (4) images that can be automatically cropped and segmented.

We also studied three **preprocessing strategies** per image (non-preprocessed, cropped, and segmented). We found that preprocessing consistently increased classification accuracy for all species and image types compared to the original non-preprocessed images. For those, the classifier is trained on a lot of potentially misleading background information. Removing vast parts of this background, through either cropping or segmentation, improved classification accuracy in all cases. In general, classification accuracy is substantially increased upon cropping, since the CNN features encode more information about the leaves, not the background. We found only slight increases in accuracy for images with natural background for segmented compared to cropped images. For images with plain background, the accuracy even decreases upon segmentation. This is induced since parts of compound leaves were accidentally removed during the segmentation especially in cases of delicate leaflets. This is true for species such as *Sanguisorba minor*, *Hippocrepis comosa*, and *Pimpinella saxifraga*, where the segmented images with plain background contained less visual information of the leaves compared to images with natural background. Against our expectation, the segmentation of images with natural background results in a very minor beneficial effect on species recognition (Fig. 4). This conclusion, however, holds only for feature classification based on CNNs, as they are superior in handling background information, when compared to handcrafted features. The images with natural background may contain more species related information such as leaf attachment to the stem, and the stem itself. The CNN features possibly encode the relative size of the object by comparing it with its background. Also, the common perspective in which leaf images of certain species are acquired is taken into account from the leaves’ natural surroundings [32].

Theoretically, it is possible to combine multiple images from the same observation in the recognition process, which could further improve the accuracy, as shown for a different dataset by [33]. We expect that the combination of different image types would also benefit the classification accuracy within our dataset. The scope of this work, however, is to reveal the most effective way of dataset acquisition.

Our discussion so far compared image types and preprocessing strategies solely based on classification accuracy. However, various combinations of methods differ only marginally from each other and it is reasonable to also consider the effort related to the acquisition process and the preprocessing of an image. Therefore we defined the accuracy-effort gain

1 $$\begin{aligned} G_i = \dfrac{a_{i}-a_{b}}{a_{b}} \dfrac{e_{b}}{e_{i}-e_{b}} \end{aligned}$$

relating obtained accuracy to the manual effort. In Eq. 1, $a_i$ represents the achieved classification accuracy using the *i*th combination of image type and preprocessing strategy and $e_i$ is the manual effort necessary to create an image of this combination. $a_b$ and $e_b$ correspond to accuracy and effort of the baseline scenario, i.e., imaging the leaf top sides in front of a natural background and applying no further preprocessing.

**Fig. 8:**
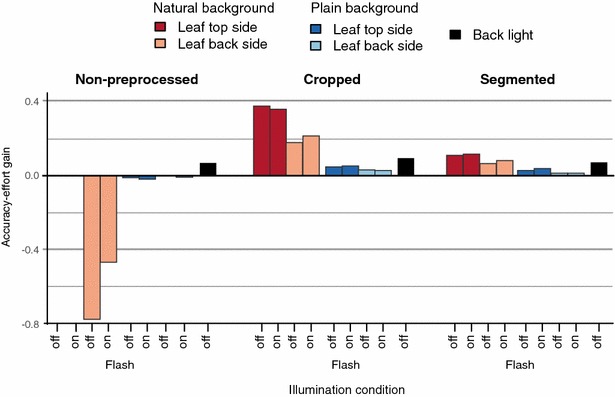
Accuracy-effort gain for the five image types and three preprocessing strategies relative to the baseline combination, i.e., non-preprocessed leaf top side images with natural background

Figure 8 indicates that the solution with optimal accuracy-effort gain is to take a top side image of a leaf with natural background and to crop it with a simple bounding box. Comparing the manual effort during image acquisition and no preprocessing, only back light images yield a positive effect on the accuracy-effort gain. Any other effort during image acquisition, e.g., by imaging the back side of a leaf or using a plain background, does not sufficiently improve the classification accuracy over the baseline. Comparing preprocessing strategies, the highest positive impact on the accuracy-effort gain is achieved by cropping. Realized by drawing a bounding box around the object of interest, cropping is a comparably simple task and required only 6.8 s per image on average in our experiments. It notably improved the classification accuracy for all image types (cp. Fig. 4). Hence, we consider cropping the most effective type of manual effort.

## Conclusion

While high accuracy is clearly the foremost aim in classification approaches, acquiring sufficiently large training datasets is a substantial investment. We argue that researchers should consider carefully how to spend available resources. Summarizing our findings in the light of human effort during image acquisition and further processing, we found that it is very useful to crop images but not to segment them. Image segmentation is a difficult and time-consuming task in particular for images with natural background. Our results show that within the used framework, this expensive step can be replaced by the much simpler but similarly effective cropping. Against our expectation, we also found no evidence that imaging leaves on plain background yields higher classification accuracy. We considered a leafs back side to be more discriminative than its top side. However, our results suggest that back side images do not yield higher accuracy but rather require considerably more human effort due to a much more challenging acquisition process. In conclusion, the most effective, non-destructive way to record herbaceous leaves in the field is taking leaf top side images and cropping them to leaf boundaries. When destructive acquisition is permissible, the back light perspective after plucking the leaf yields the best overall result in terms of recognition accuracy.
